# Phenoxymethylpenicillin Versus Amoxicillin for Infections in Ambulatory Care: A Systematic Review

**DOI:** 10.3390/antibiotics7030081

**Published:** 2018-09-04

**Authors:** Philip Lawrence Skarpeid, Sigurd Høye

**Affiliations:** 1 Faculty of Medicine, University of Oslo, 0318 Oslo, Norway; pls@live.no; 2 The Antibiotic Centre for Primary Care, Department of General Practice, Institute of Health and Society, University of Oslo, 0318 Oslo, Norway

**Keywords:** phenoxymethylpenicillin, amoxicillin, antibiotics, resistance, treatment, ambulatory care, primary health care

## Abstract

Most antibiotics are prescribed in primary care, and commonly for respiratory tract infections (RTIs). Narrow-spectrum phenoxymethylpenicillin is the antibiotic of choice for RTIs in the Scandinavian countries, while broader spectrum amoxicillin is used in most other European countries. This review summarizes the knowledge of the effect of phenoxymethylpenicillin versus amoxicillin for infections treated in ambulatory care. We searched PubMed/Medline and Embase for trials comparing the clinical effect of phenoxymethylpenicillin and amoxicillin. The Norwegian Knowledge Centre for the Health Services’ checklist was used to assess risk of bias. In total, 1687 studies were identified, and 18 of these fulfilled the inclusion criteria. One additional study was found as a reference. The randomized controlled trials revealed no significant differences in clinical effect in acute sinusitis (three RCTs), GAS tonsillitis (11 RCTs) and Lyme borreliosis (two RCTs). One RCT on community-acquired pneumonia found amoxicillin to be superior, while the results were conflicting in the two RCTs on acute otitis. The results suggest that non-Scandinavian countries should consider phenoxymethylpenicillin as the treatment of choice for RTIs because of its narrower spectrum. More studies should be conducted on the clinical effect of phenoxymethylpenicillin versus amoxicillin for acute otitis and lower RTIs.

## 1. Introduction

Use of broad-spectrum antibiotics increases the risk of antimicrobial resistance. To meet the global challenge of antimicrobial resistance, the use of antibiotics, and especially the use of broad-spectrum antibiotics, should be kept low throughout the world. 

Most antibiotics are prescribed outside of hospitals and nursing homes, and mostly by general practitioners [1]. Of these prescriptions, approximately 60% are for respiratory tract infections (RTIs). Norway is in a position where narrow-spectrum phenoxymethylpenicillin is the recommended treatment for most RTIs when antibiotics are warranted, as well as for erysipelas and erythema migrans [2]. As a consequence, phenoxymethylpenicillin makes up as much as 26% of the antibiotics sold in Norwegian pharmacies (exclusive of methenamin), measured in defined daily doses (DDD) [3]. Sweden and Denmark have similar recommendations, and in a 2012 review of outpatient antibiotics use in 33 European countries [4], these three Scandinavian countries were the only countries where narrow-spectrum penicillins accounted for more than half of the penicillin consumption. In other European countries, the broader spectrum antibiotic amoxicillin with or without enzyme inhibitor (co-amoxiclav) is the recommended first line treatment for various RTIs when antibiotics are warranted, and the proportion of narrow-spectrum penicillin of all antibiotics is generally low (Germany 6%, Spain 0,5%, UK 4%) [4,5]. 

The proportion of phenoxymethylpenicillin use compared with more broad-spectrum antibiotics is declining, and phenoxymethylpenicillin has been labelled “a forgotten antibiotic” [6]. In the search for measures to increase the relative use of narrow-spectrum antibiotics, it is important to explore the reasons behind the difference in recommendations for the most common infections treated with antibiotics. The aim of this review is to summarize the knowledge of clinical effect of phenoxymethylpenicillin versus amoxicillin for any diagnosis in ambulatory care.

## 2. Results

An overview of the different studies included in this review [7,8,9,10,11,12,13,14,15,16,17,18,19,20,21,22,23,24,25] is given in Table 1. Two controlled trials and 17 randomized controlled trials were included. Below follows a summarization of the results for the different studies, grouped by diagnosis. 

### 2.1. Group A Streptococci (GAS) Tonsillitis

Eleven studies [7,8,9,10,11,12,13,14,15,16] have been published comparing clinical effect of phenoxymethylpenicillin and amoxicillin in the treatment of GAS. All but one [15] are randomized controlled trials. Amoxicillin came into medical use around 1972 [26], and the first studies from 1974 and onward aimed to compare the effectiveness and adverse effects of this new drug with that of phenoxymethylpenicillin [7,8,9], or to compare these two drugs with even newer antibiotics [10]. The RCTs from 1993 and onward aimed to investigate the effect of amoxicillin administered once [11,14,16,17] or twice [3,12] daily, and/or a six to seven days course [12,13,17], compared with the conventional 10 days course of phenoxymethylpenicillin administered three or four times daily. The definition of clinical success varied between the studies. No significant differences in cure rates were found in any of the studies. The authors typically conclude that amoxicillin is equivalent to phenoxymethylpenicillin, even when amoxicillin is administered once or twice daily for six to seven days. Due to better compliance with easier regimens [12,16], the relative narrow-spectrum and low price of amoxicillin compared with other antibiotics that may be administered once daily, and the fact that amoxicillin already is in widespread use for respiratory tract infections [12], the authors argue that amoxicillin is a reasonable treatment alternative for GAS infections.

One single non-randomized trial was identified [15]. The authors aimed to reexamine the efficacy of the two drugs. Choice of antibiotic therapy was according to the physician’s discretion. At the follow-up visit after about 10 days, the cure rate was significantly highest in the amoxicillin group. Phenoxymethylpenicillin was administered twice daily, as opposed to all other studies, where the drug was administered at least thrice daily. The authors suspect that the difference may be due to low compliance caused by the poor taste of phenoxymethylpenicillin, and argue that differences in compliance are just as important as differences in pharmacodynamics when choosing an antibiotic regimen.

### 2.2. Acute Sinusitis

The aim of all the three studies [18,19,20] on acute sinusitis was to investigate the effect of the two antibiotics compared with placebo. All studies used some form of imaging in addition to signs and symptoms to confirm the diagnosis of sinusitis. In the oldest study [18], acute sinusitis was confirmed by the presence of fluid levels or total opacification at computed tomography (CT) scans. The same author later investigated the treatment effect in patients where CT scans showed mucosal thickening [19]. In the most recent study [20], a doxycyclin group was included, and ultrasound was used to detect presence of fluid in the maxillary sinuses. No significant differences in cure rates between phenoxymethylpenicillin and amoxicillin were found in any of the studies. However, all studies were underpowered, and a non-significant difference in symptom duration (amoxicillin: nine days, phenoxymethylpenicillin: 11 days) was found in the first study. Relatively high doses of phenoxymethylpenicillin were used in all studies, and the author of the two first studies argue that treating acute sinusitis with phenoxymethylpenicillin in high doses seems reasonable. 

### 2.3. Acute Otitis Media

Two studies on acute otitis media were identified. The first study [21] aimed to compare the efficacy of the then new antibiotic erythromycin (with or without trisulfapyrimidine) with the traditional drugs phenoxymethylpenicillin and amoxicillin. Cure rates at day 10 differed significantly between amoxicillin and the other antibiotics combined. Only for *S. pneumoniae* infections cure rate numbers were given (amoxicillin 92%, phenoxymethylpenicillin 75%), while for *H. influenzae* infections, amoxicillin was grouped with erythromycin plus trisulfapyrimidine (cure rate 94%), and phenoxymethylpenicillin was grouped with erythromycin (cure rate 67%). The authors suspected that the superiority of amoxicillin was due to the less uniform gastrointestinal absorption of phenoxymethylpenicillin, and failure to achieve great enough concentrations of the drug in middle ear exudate. In the second study [22], the phenoxymethylpenicillin dosage was considerably higher. This study aimed to determine the necessity of using broad-spectrum antibiotics rather than the traditional first choice of phenoxymethylpenicillin. At follow-up examination at day 10, the cure rate was highest in the phenoxymethylpenicillin group, while the cure rates were similar in both groups at day 24. As the two drugs were equally effective even though *H. influenzae* was detected in a substantial portion of the patients, the authors argue that phenoxymethylpenicillin is the drug of choice for the treatment of acute otitis media in out-patients.

### 2.4. Lyme Borreliosis

We identified two studies on Lyme borreliosis [23,24]. In a non-randomized trial [23], patients with the rare diagnosis of borrelial lymphocytoma were treated with amoxicillin, phenoxymethylpenicillin, doxycyclin or azithromycin. No statistical tests were performed, as the treatment groups were small and heterogenous. Due to treatment failure in two of 19 patients treated with phenoxymethylpenicillin, the authors suggest that other antibiotics may be superior for this condition. In a recent RCT [24], patients with erythema migrans diagnosed in primary care were assigned to either amoxicillin, phenoxymethylpenicillin or doxycyclin. As no clinical differences were found between the groups, the authors suggest that phenoxymethylpenicillin should be the drug of choice for solitary erythema migrans.

### 2.5. Pneumonia

One non-inferiority RCT was identified where phenoxymethylpenicillin was tested against amoxicillin for the treatment of community-acquired pneumonia [25]. Patients were recruited in primary care, and the diagnosis of pneumonia was radiologically confirmed. At day 14 the patients were evaluated. In the per protocol analysis no significant difference in cure rates was found between the treatment groups, but in the intention-to-treat analysis, amoxicillin was found to be 28,6% superior to phenoxymethylpenicillin (95% CI, 7.3–58.1%; *p* = 0.009 for superiority). The study was underpowered, as only 43 of the required sample size of 210 patients were recruited. 

## 3. Discussion

### 3.1. Summary of Main Results

According to our results, 19 studies have been performed where phenoxymethylpenicillin has been tested directly against amoxicillin for the treatment of any diagnosis in ambulatory care. More than half of the studies were performed on the treatment of GAS tonsillitis. 

Both for GAS tonsillitis, acute sinusitis and erythema migrans, no significant differences in effect were found between the two antibiotics in randomized controlled trials. For acute otitis media, the most recent study found no significant difference between the two types of antibiotics, while the six-year-older study showed a significant difference in favour of amoxicillin. In the single trial on community-acquired pneumonia, no significant differences were found in the per protocol analysis. However, amoxicillin was found to be superior to phenoxymethylpenicillin in the intention-to-treat analysis. 

### 3.2. Strengths and Limitations

We performed a systematic literature search in two databases, which strengthens the probability for including the relevant literature. Only studies reporting clinical data from ambulatory care were included in the review, as our aim was to compare clinical effects relevant for patients and practitioners in primary care. Some of the studies had bacteriological eradication as a primary outcome, and clinical cure as secondary outcome. Microbiological data and data from hospital settings might have strengthened the review. Studies comparing phenoxymethylpenicillin or amoxicillin solely with other antibiotics were not included. Also, studies where amoxicillin was given in combination with clavulanic acid (co-amoxiclav) were excluded, as our aim was to compare the two treatment options most commonly recommended in treatment guidelines in primary care. As such, the results cannot say what is the best of all treatments for the various diagnosis in primary care, only which of the two treatment options that shows best clinical effect when compared in a trial. 

The diagnostic criteria and the definitions of clinical success varied greatly between the different studies, and in some of the oldest studies, success was defined as a combination of clinical cure and negative pharyngeal cultures. We have reported what the authors themselves define as clinical success. In addition, there was large variation in the antibiotic dosages and durations of courses used in the different studies. This heterogenity makes it hard to compare the results from the different studies within each diagnosis group. 

All studies were performed in high income countries. This weakens the ability to generalize the findings to low and middle-income countries.

### 3.3. Comparison with Other Literature

For GAS tonsillitis, there is a general agreement between guidelines that phenoxymethylpenicillin is the recommended treatment when an antibiotic is warranted. This is in line with the results in this review, and also with the fact that penicillin-resistant Group A streptococci has not been discovered [27].

For acute otitis media in children, there is disagreement between guidelines. Scandinavian guidelines [2,28] recommend phenoxymethylpenicillin when an antibiotic is warranted. The much-used clinical resource UpToDate acknowledges that there is no evidence to support a particular antibiotic regimen over another, and recommends amoxicillin due to its safety, inexpensiveness and narrow microbiologic spectrum [29]. In England, The National Institute for Health and Care Excellence (NICE) also recommends amoxicillin as first line treatment, based on evidence, experience and resistance data, and “because this is current practice for antibiotic treatment in children with acute otitis media” [30]. The evidence summarized in this review does not support amoxicillin being preferred to phenoxymethylpenicillin in the treatment of acute otitis media in children.

For acute sinusitis, UpToDate recommends amoxicillin-clavulate as first line empirical treatment due to the increasing microbial resistance to antibiotics, and argue that clinical studies on acute sinusitis may fail to differentiate between antibiotics due to the high rate of spontaneous recovery in sinusitis patients [31]*.* NICE guidelines, however, recommend phenoxymethylpenicillin as first line treatment, based on the evidence of no important differences in clinical effectiveness between classes of antibiotics [32], which is in line with the results in this review. 

UpToDate recommends doxycycline, cefuroxime or amoxicillin for the treatment of early Lyme disease/erythema migrans. This recommendation is based on trials where phenoxymethylpenicillin was not included [33]. NICE guidelines recommend doxycycline, alternatively amoxicillin or azithromycin for erythema migrans [34]. For both guidelines, the literature search ended prior to the publication of the RCT on erythema migrans included in this review [24], which concluded that phenoxymethylpenicillin, doxycycline and amoxicillin do not differ in effectiveness in the treatment of erythema migrans in primary care. Norwegian and Swedish guidelines recommend phenoxymethylpenicillin for solitary erythema migrans [2,35].

NICE guidelines recommend amoxicillin as the treatment of choice for low-severity community-acquired pneumonia [36], while Scandinavian guidelines recommend phenoxymethylpenicillin [2]. As shown in this review, only one clinical study has compared the two antibiotics. The study was performed in Spain, which has relatively high levels of penicillin-resistant *S. pneumoniae* [25]. Even though the study was heavily underpowered, significant differences in favour of amoxicillin were found in the intention-to-treat analysis. However, three out of the 14 patients in the phenoxymethylpenicillin group were lost to follow up, compared to none in the amoxicillin group, and there were no significant differences in clinical cure rates for the patients that were examined at day 14. The low number of patients in this study makes it hard to draw definitive conclusions. Similar studies have not been performed in countries with low levels of penicillin-resistant *S. pneumoniae.*

We found no relevant studies on acute bronchitis. Even though antibiotics are not recommended in the treatment of acute bronchitis, it is a major reason for antibiotic use in primary care, commonly with amoxicillin or other broad-spectrum antibiotics [37]. Also, no studies on the treatment of unspecified upper RTI were found. Acute bronchitis and unspecified upper RTI have been found to account for as much as 42% of antibiotics prescribed for RTIs [38].

Even though amoxicillin is recommended due to its relatively narrow-spectrum in several of the studies in this review, its antimicrobial spectrum is considerably broader than that of phenoxymethylpenicillin. Amoxicillin supresses enterobacteria and may give overgrowth of *Candida* and *C. difficile*, unlike phenoxymethylpenicillin [39]. 

The reason to prefer amoxicillin to phenoxymethylpenicillin as empirical treatment in primary care may be the fear of treatment failure due to penicillin-resistant *Haemophilus influenzae* or *Streptococcus pneumoniae. S. pneumoniae* is the main cause of community-acquired pneumonia, and it is also one of the most frequent bacterial causes of acute otitis media and sinusitis [2]. However, Scandinavian countries and the UK have close to identical low percentages of penicillin-resistant *S. pneumoniae* (Norway 4,4%, UK 4.9%), and this is also the case for several other European countries, for example, Germany (6.0%) and Italy (6.5%). Spain and France, on the other hand, are reportedly in a situation with much higher percentage (25,0% and 25.3% respectively) of penicillin-resistant *S. pneumoniae* [40]. We have previously demonstrated that although the antibiotic switch rate after initial prescribing of phenoxymethylpenicillin in primary care is higher than that of amoxicillin (4.1% versus 2.5%), it is still low, and may be caused by other factors than treatment failure [41].

It may seem that, in many instances, the preference of amoxicillin over phenoxymethylpenicillin is based not on clinical trials, but on historical or microbiological considerations. This review highlights that for the diagnoses of GAS tonsillitis, erythema migrans, and acute sinusitis in primary care, clinical evidence supports the recommendation of phenoxymethylpenicillin as a first line treatment. In countries where amoxicillin is the current recommended treatment for acute otitis and community-acquired pneumonia, there is a potential and a need for non-inferiority studies comparing phenoxymethylpenicillin and amoxicillin for these conditions, especially in settings with low levels of penicillin-resistant *S. pneumoniae*. 

Regarding treatment with antibiotics in primary care, the case is more often whether not to prescribe rather than what to prescribe. The goal must be to avoid unnecessary antibiotics use altogether, but in order to minimize both societal and individual adverse effects, it is better to prescribe unnecessary phenoxymethylpenicillin than to prescribe unnecessary amoxicillin.

## 4. Materials and Methods

### 4.1. Basis of Knowledge

**Figure 1:**
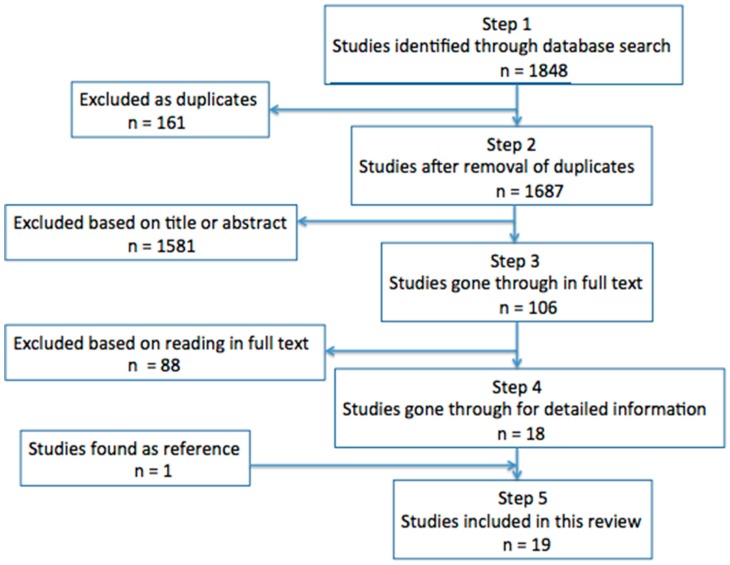
Identification of the included studies. “n” is a combined number of studies identified through both the initial and the updated search.

We searched for relevant literature in both PubMed/Medline and Embase, with no time limits. The search was performed on the 24th of May 2016, with an updated search on the 18th of June 2018 (Figure 1). One of the authors (PLS) went through titles and abstracts of all identified articles, and included studies based on predefined inclusion and exclusion criteria. In case of uncertainty, full text versions were obtained and read through. If still in doubt on whether to include a study, the authors (PLS and SH) discussed until a decision was agreed upon. The reference lists in the included studies were examined for studies that fitted the predefined inclusion criteria. All included articles were read in full text. 

### 4.2. Inclusion Criteria

All studies published where the clinical effect of phenoxymethylpenicillin and amoxicillin, given per os, have been compared, in either a randomized controlled trial or solely controlled trial, as treatment for any diagnosis in an ambulatory care setting. 

### 4.3. Exclusion Criteria

Trials performed in a hospital setting, trials concerning dental health, trials reporting only microbiological and not clinical effect, and trials where amoxicillin was given in combination with clavulanic acid.

### 4.4. Quality Assessment

The quality of the included randomised controlled trials (RCTs) was assessed by using the first six criteria from a checklist designed for RCTs, provided by the Norwegian Knowledge Centre for the Health Services [42]. The quality was considered strong if five or six of the criteria were met, moderate if three or four of the criteria were met, and low if one, two or none of the criteria were met.

The quality of the included controlled trials (CTs) was assessed using five of the six criteria used for the RCTs. The quality of these studies was considered strong if four or five of the criteria were met, moderate if two or three of the criteria were met, and low if one or none of the criteria were met. 

### 4.5. Seach Strings

The search in PubMed/Medline contained the Mesh terms “Amoxicillin” and “Penicillin v”. The limitations for the search were as follows: Filters: Humans; Languages: Danish; English; Norwegian; Swedish. The search yielded a total of 202 hits in 2016, with an additional 14 hits in 2018. The search in Embase contained the subheadings Amoxicillin/ and Penicillin V/. The limitations for the search were as follows: Human, English, Norwegian, Danish, Swedish. This search yielded a total of 1499 hits in 2016, and an additional 133 hits in 2018. The search strings are available in the supplementary material.

## Figures and Tables

**Table 1:** Overview of the included studies.

Lead Author (Reference)	Year, Country	Design, Population	Age in Years	Daily Dose (days) PcV-Amoxicillin	Clinical Outcome PcV-Amoxicillin	Signific-ance	Quality
**GAS**							
Breese, B.B. [7]	1974, USA	RCT, 159	“Children”	375 mg (10)–375 mg (10)	Cure rate after five weeks: 80.8–76.6%	NSD	Medium
Stillerman, M. [8]	1974, USA	RCT, 112	“Children”	375 mg (10)–375 mg (10)	Cure rate within 72 h: 91–89%	*	Medium
Breese, B.B. [9]	1977, USA	RCT, 172 ^1^	1–17	375–750 mg (10)–375–750 mg (10)	Cure rate after six weeks: 87.9–90.6%	NSD	Medium
Pankey, G. A. [10]	1981, USA	RCT, 83 ^2^	6–40	1000 mg (10)–750 mg (10)	Cure rate at day 13–14: 100–100%	NSD	Low
Shvartzman, P. [11]	1993, Israel	RCT, 157	>3	750–1000 mg (10)–750 mg (10)	Days off school/work: 139 days for 64 patients–100 days for 57 patients	NSD	Medium
Peyramond, D. [12]	1996, France	RCT, 342	>15	1980 mg (10)–2000 mg (6)	Cure rate at end of treatment: 96.5–96.4%	NSD	High
Cohen, R. M.D. [13]	1996, France	RCT, 321	3–15	45 mg/kg (10)–50 mg/kg (6)	Cure rate: 89.0–90.8%	NSD	Medium
Feder, H.M., Jr. [14]	1999, USA	RCT, 152	4–18	750 mg (10)–750 mg (10)	Cure rate after 18–24 h: 90–90%	NSD	Medium
Curtin-Wirt, C. [15]	2003, USA	CT, 389	0–20	35 mg/kg–1000 mg (10)–35 mg/kg–1000 mg (10)	Cure rate at day 10 +/− 4: 73–84%	Amoxicillin superior, *p* = 0.03	High
Lennon, D. R. [16]	2008, NZ	RCT, 353	5–12	500–1000 (10)–750–1500 (10)	“No difference in clinical symptom resolution”	*	High
Pichichero, M. E. [17]	2008, USA	RCT, 579	0–12	40 mg/kg (10)–475–775 mg (7)	Cure rate: 91.9–86.1%	NSD	High
**Acute Sinusitis**							
Lindbaek, M. [18]	1996, Norway	RCT, 130 ^3^	16–74	3960 mg (10)–1500 mg (10)	Cure rate at day 10: 82.1–88.6%	NSD	High
Lindbaek, M. [19]	1998, Norway	RCT, 70 ^3^	16–83	3960 mg (10)–1500 mg (10)	Cure rates at day 10: 75–77%	NSD	Medium
Varonen, H. [20]	2003, Finland	RCT, 150 ^3,4^	18–75	3000 mg (7)–1500 mg (7)	Cure rate at day 14–16: 81–78%	NSD	High
**Acute Otitis**							
Howard, J. E. [21]	1976, USA	RCT, 383 ^5^	0–5	50 mg/kg (10)–30 mg/kg (10)	Cure rate at day 10: 75–92%	Amoxicillin superior, *p* < 0.05	High
Puhakka, H. [22]	1982, Finland	RCT, 65	0–9	75–80 mg/kg (10)–40 mg/kg (10)	Cure rate at day 10 and day 24: 44–32%, 88–87%	NSD	Medium
**Lyme Borreliosis**							
Strle, F. [23]	1996, Slovenia	CT, 65 ^4,6^	1–72	3000 mg (14)–3000 mg (14)	Median duration of lymphocytoma: 2 weeks–1.5 weeks.	*	Medium
Eliassen K.E. [24]	2018, Norway	RCT, 188 ^4^	18–85	3900 mg (14)–1500 mg (14)	Median duration of erythema migrans: 14 days–13 days	NSD	High
**Pneumonia**							
Llor, C. [25]	2017, Spain	RCT, 36	18–75	3200 mg (10)–3000 mg (10)	Cure rate at day 14: 71.4–100% (ITT) 90.9–100% (PP)	ITT: Amoxicillin superio, *p* = 0.009. PP: NSD	High

RCT: Randomized Controlled Trial CT: Controlled Trial GAS: Group A Streptococcci S: Significant NS: Non-Significant NSD: Non Significant Difference NZ: New Zealand PcV: Penicillin V PP: Per protocol-analysis ITT: Intention to treat-analysis. ^1^: Erythromycin-group included. ^2^: Bacampicillin-group included. ^3^: Placebo-group included. ^4^: Doxycycline-group included. ^5^: Erythromycin- and erythromycin plus trisulfapyrimidines-groups included. ^6^: Azithromycin-group included. * Statistical tests not performed.

## Supplementary Materials

The following are available online at http://www.mdpi.com/2079-6382/7/3/81/s1, Text document S1: Search strings.
