# Supplementary material for: Phenoxymethylpenicillin Versus Amoxicillin for Infections in Ambulatory Care: A Systematic Review
Source: Antibiotics (Basel). 2018 Sep 4;7(3):81. doi: 10.3390/antibiotics7030081 (PMC6163205; doi:10.3390/antibiotics7030081)
Supplement: Supplementary file 1 [file antibiotics-07-00081-s001.pdf]

antibiotics

Supplementary Material

Phenoxymethylpenicillin Versus Amoxicillin for Infections in Ambulatory Care: A Systematic Review

Philip Lawrence Skarpeid [1], Sigurd Høye [2,*]

[1] Faculty of Medicine, University of Oslo, Oslo 0318 Norway; pls@live.no
[2] The Antibiotic Centre for Primary Care, Department of General Practice, Institute of Health and Society, University of Oslo, 0318 Norway
* Correspondence: sigurd.hoye@medisin.uio.no; Tel.: +47-40-45-17-47

Received: 06 July 2018; Accepted: 29 August 2018; Published: date

Search strings:

PubMed/Medline search string:

Search (((penicillin v) OR ((((((((((((((((((((Phenoxymethylpenicillin) OR Fenoxymethylpenicillin) OR Penicillin, Phenoxymethyl) OR Phenoxymethyl Penicillin) OR Beromycin) OR Berromycin, Penicillin) OR Penicillin Berromycin) OR Beromycin, Penicillin) OR Penicillin Beromycin) OR Betapen) OR Pen VK) OR Penicillin VK) OR Penicillin V Sodium) OR Sodium, Penicillin V) OR V Sodium, Penicillin) OR V-Cillin K) OR V Cillin K) OR VCillin K) OR Vegacillin) OR Apocillin) OR Penicillin V Potassium) OR Potassium, Penicillin V))) AND ((amoxicillin) OR ((((((((((((((((((((((((((((Amoxycillin) OR Amoxicillin Trihydrate) OR Trihydrate, Amoxicillin) OR Hydroxyampicillin) OR Amoxicillin Monopotassium Salt) OR Amoxicillin Sodium) OR Sodium, Amoxicillin) OR Amoxicillin Monosodium Salt) OR Amoxicillin, (R*)-Isomer) OR Amoxicillin Anhydrous) OR Anhydrous, Amoxicillin) OR Amoxicilline) OR BRL-2333) OR BRL 2333) OR BRL2333) OR Clamoxyl) OR Penamox) OR Clamoxyl G.A.) OR G.A., Clamoxyl) OR Pfizer Brand of Amoxicillin Sodium Salt) OR SmithKline Beecham Brand of Amoxicillin Sodium Salt) OR Clamoxyl parenteral) OR parenteral, Clamoxyl) OR Polymox) OR Trimox) OR Wymox) OR Actimoxi) OR Clariana Brand of Amoxicillin) OR Amoxicillin Clariana Brand) OR Amoxil))

Embase search string:

penicillin V/
OR
Phenoxymethylpenicillin.mp. [mp=title, abstract, heading word, drug trade name, original title, device manufacturer, drug manufacturer, device trade name, keyword]
OR
Fenoxymethylpenicillin.mp. [mp=title, abstract, heading word, drug trade name, original title, device manufacturer, drug manufacturer, device trade name, keyword]
OR
Penicillin, Phenoxymethyl.mp. [mp=title, abstract, heading word, drug trade name, original title, device manufacturer, drug manufacturer, device trade name, keyword]
OR
Phenoxymethyl Penicillin.mp. [mp=title, abstract, heading word, drug trade name, original title, device manufacturer, drug manufacturer, device trade name, keyword]
OR
Beromycin, Penicillin.mp. [mp=title, abstract, heading word, drug trade name, original title, device manufacturer, drug manufacturer, device trade name, keyword]
OR

Berromycin, Penicillin.mp. [mp=title, abstract, heading word, drug trade name, original title, device manufacturer, drug manufacturer, device trade name, keyword]

OR

Penicillin Berromycin.mp. [mp=title, abstract, heading word, drug trade name, original title, device manufacturer, drug manufacturer, device trade name, keyword]

OR

Beromycin, Penicillin.mp. [mp=title, abstract, heading word, drug trade name, original title, device manufacturer, drug manufacturer, device trade name, keyword]

OR

Penicillin Beromycin.mp. [mp=title, abstract, heading word, drug trade name, original title, device manufacturer, drug manufacturer, device trade name, keyword]

OR

Betapen.mp. [mp=title, abstract, heading word, drug trade name, original title, device manufacturer, drug manufacturer, device trade name, keyword]

OR

Pen VK.mp. [mp=title, abstract, heading word, drug trade name, original title, device manufacturer, drug manufacturer, device trade name, keyword]

OR

Penicillin VK.mp. [mp=title, abstract, heading word, drug trade name, original title, device manufacturer, drug manufacturer, device trade name, keyword]

OR

Penicillin V Sodium.mp. [mp=title, abstract, heading word, drug trade name, original title, device manufacturer, drug manufacturer, device trade name, keyword]

OR

Sodium, Penicillin V.mp. [mp=title, abstract, heading word, drug trade name, original title, device manufacturer, drug manufacturer, device trade name, keyword]

OR

V Sodium, Penicillin.mp. [mp=title, abstract, heading word, drug trade name, original title, device manufacturer, drug manufacturer, device trade name, keyword]

OR

V-Cillin K.mp. [mp=title, abstract, heading word, drug trade name, original title, device manufacturer, drug manufacturer, device trade name, keyword]

OR

V Cillin K.mp. [mp=title, abstract, heading word, drug trade name, original title, device manufacturer, drug manufacturer, device trade name, keyword]

OR

VCillin K.mp. [mp=title, abstract, heading word, drug trade name, original title, device manufacturer, drug manufacturer, device trade name, keyword]

OR

Vegacillin.mp. [mp=title, abstract, heading word, drug trade name, original title, device manufacturer, drug manufacturer, device trade name, keyword]

OR

Apocillin.mp. [mp=title, abstract, heading word, drug trade name, original title, device manufacturer, drug manufacturer, device trade name, keyword]

OR

Penicillin V Potassium.mp. [mp=title, abstract, heading word, drug trade name, original title, device manufacturer, drug manufacturer, device trade name, keyword]

OR

Potassium, Penicillin V.mp. [mp=title, abstract, heading word, drug trade name, original title, device manufacturer, drug manufacturer, device trade name, keyword]

AND

amoxicillin/

OR

Amoxycillin.mp. [mp=title, abstract, heading word, drug trade name, original title, device manufacturer, drug manufacturer, device trade name, keyword]

OR

Amoxicillin Trihydrate.mp. [mp=title, abstract, heading word, drug trade name, original title, device manufacturer, drug manufacturer, device trade name, keyword]

OR

Trihydrate, Amoxicillin.mp. [mp=title, abstract, heading word, drug trade name, original title, device manufacturer, drug manufacturer, device trade name, keyword]

OR

Hydroxyampicillin.mp. [mp=title, abstract, heading word, drug trade name, original title, device manufacturer, drug manufacturer, device trade name, keyword]

OR

Amoxicillin Monopotassium Salt.mp. [mp=title, abstract, heading word, drug trade name, original title, device manufacturer, drug manufacturer, device trade name, keyword]

OR

Amoxicillin Sodium.mp. [mp=title, abstract, heading word, drug trade name, original title, device manufacturer, drug manufacturer, device trade name, keyword]

OR

Sodium, Amoxicillin.mp. [mp=title, abstract, heading word, drug trade name, original title, device manufacturer, drug manufacturer, device trade name, keyword]

OR

Amoxicillin Monosodium Salt.mp. [mp=title, abstract, heading word, drug trade name, original title, device manufacturer, drug manufacturer, device trade name, keyword]

OR

Amoxicillin Anhydrous.mp. [mp=title, abstract, heading word, drug trade name, original title, device manufacturer, drug manufacturer, device trade name, keyword]

OR

Anhydrous, Amoxicillin.mp. [mp=title, abstract, heading word, drug trade name, original title, device manufacturer, drug manufacturer, device trade name, keyword]

OR

Amoxicilline.mp. [mp=title, abstract, heading word, drug trade name, original title, device manufacturer, drug manufacturer, device trade name, keyword]

OR

BRL-2333.mp. [mp=title, abstract, heading word, drug trade name, original title, device manufacturer, drug manufacturer, device trade name, keyword]

OR

BRL 2333.mp. [mp=title, abstract, heading word, drug trade name, original title, device manufacturer, drug manufacturer, device trade name, keyword]

OR

BRL2333.mp. [mp=title, abstract, heading word, drug trade name, original title, device manufacturer, drug manufacturer, device trade name, keyword]

OR

Clamoxyl.mp. [mp=title, abstract, heading word, drug trade name, original title, device manufacturer, drug manufacturer, device trade name, keyword]

OR

Penamox.mp. [mp=title, abstract, heading word, drug trade name, original title, device manufacturer, drug manufacturer, device trade name, keyword]

OR

Pfizer Brand of Amoxicillin Sodium Salt.mp. [mp=title, abstract, heading word, drug trade name, original title, device manufacturer, drug manufacturer, device trade name, keyword]

OR

SmithKline Beecham Brand of Amoxicillin Sodium Salt.mp. [mp=title, abstract, heading word, drug trade name, original title, device manufacturer, drug manufacturer, device trade name, keyword]

OR

Clamoxyl parenteral.mp. [mp=title, abstract, heading word, drug trade name, original title, device manufacturer, drug manufacturer, device trade name, keyword]

OR

parenteral, Clamoxyl.mp. [mp=title, abstract, heading word, drug trade name, original title, device manufacturer, drug manufacturer, device trade name, keyword]

OR

Polymox.mp. [mp=title, abstract, heading word, drug trade name, original title, device manufacturer, drug manufacturer, device trade name, keyword]

OR

Trimox.mp. [mp=title, abstract, heading word, drug trade name, original title, device manufacturer, drug manufacturer, device trade name, keyword]

OR

Wymox.mp. [mp=title, abstract, heading word, drug trade name, original title, device manufacturer, drug manufacturer, device trade name, keyword]

OR

Actimoxi.mp. [mp=title, abstract, heading word, drug trade name, original title, device manufacturer, drug manufacturer, device trade name, keyword]

OR

Clariana Brand of Amoxicillin.mp. [mp=title, abstract, heading word, drug trade name, original title, device manufacturer, drug manufacturer, device trade name, keyword]

OR

Amoxicillin Clariana Brand.mp. [mp=title, abstract, heading word, drug trade name, original title, device manufacturer, drug manufacturer, device trade name, keyword]

OR

Amoxil.mp. [mp=title, abstract, heading word, drug trade name, original title, device manufacturer, drug manufacturer, device trade name, keyword]
